# Evaluation of Ocular Versions in Graves' Orbitopathy: Correlation between the Qualitative Clinical Method and the Quantitative Photographic Method

**DOI:** 10.1155/2020/9758153

**Published:** 2020-07-31

**Authors:** Cristiane de Almeida Leite, Thaís de Sousa Pereira, Jeane Chiang, Allan C. Pieroni Gonçalves, Mário L. R. Monteiro

**Affiliations:** Laboratory of Investigation in Ophthalmology (LIM 33), Division of Ophthalmology, University of São Paulo Medical School, São Paulo, Brazil

## Abstract

**Purpose:**

To assess the agreement between the qualitative clinical method and the quantitative photographic method of evaluating normal and abnormal ocular versions in patients with inactive Graves' orbitopathy (GO).

**Methods:**

Forty-two patients with inactive GO had their ocular versions evaluated clinically according to three categories: normal, moderate alterations (−1 or −2 hypofunction), and severe alterations (−3 or −4 hypofunction). The subjects were photographed in the 9 positions of gaze, and the extent (mm) of eye movement in each position was estimated using Photoshop^®^ and ImageJ and converted into degrees with a well-established method. The agreement between the two methods (qualitative vs. quantitative) for classifying ocular versions as normal or abnormal was assessed.

**Results:**

The mean quantitative measurements of versions were significantly different for each clinical category (normal, moderate alterations, and severe alterations) in the following five positions: abduction, adduction, elevation in abduction, elevation, and elevation in adduction (*p* < 0.001). No such pattern was observed for the three infraversion positions (depression in abduction, *p*=0.573; depression, *p*=0.468; depression in adduction, *p*=0.268).

**Conclusion:**

The agreement was strong between the quantitative photographic method and the qualitative clinical method of classifying ocular versions, especially in lateral and supraversions, which are typically affected in GO. Digital photography is recommended for the assessment of ocular versions due to its practicality, suitability for telemedicine applications, and ease of monitoring during follow-up. This trial is registered with NCT03278964.

## 1. Introduction

Assessment of ocular versions is an essential part of the study of extrinsic ocular motility, helping in the diagnosis and treatment of eye movement disorders, especially incomitant, restrictive, and paralytic strabismus [1].

Evaluations during clinical examination are usually qualitative. The patient is instructed to follow an object presented by the examiner, from the primary position to secondary and tertiary positions of gaze. For each muscle involved, versions are graded from −1 to −4 for hypofunction and from +1 to +4 for hyperfunction. Due to high interobserver variability and standardization errors, the method is heavily dependent on examiner experience [2, 3]. To circumvent this problem, quantitative measuring methods with objective scales have been proposed [4–11].

Quantitative version assessments can be made with kinetic methods (the patient following a moving target) or static methods (measuring the angle of movement in a given position of gaze) [5]. Examples of the former are the limbus test [8], the lateral version light-reflex test [9], and the use of ophthalmic devices such as perimeters [10]. The latter includes the use of Hess and Lancaster screens [5].

In 2014, Lim and colleagues described a modified limbus test, evaluating versions based on photographs taken in the cardinal positions of gaze [11]. This low-cost method has proven to be reproducible and easily implemented in clinical practice. In this study, we evaluated the agreement between the qualitative clinical method and the quantitative photographic method of assessing ocular versions in a sample of patients with Graves' orbitopathy (GO) with different degrees of ocular version abnormalities.

## 2. Methods

This prospective and comparative study was conducted at a hospital-based outpatient referral ophthalmology service in São Paulo, Brazil. The study protocol complied with the tenets of the latest revision of the Declaration of Helsinki and was approved by the Institutional Review Board of the University of São Paulo Medical School. All participants gave their informed written consent. Between January 2015 and November 2018, 42 patients in the inactive phase of GO were studied. GO was quantified with the Clinical Activity Score (CAS) [12]. Patients with CAS <3 for at least 6 months and time of onset of GO >2years were considered to have inactive disease.

The inclusion criteria were as follows: (i) diagnosis of GO in the inactive phase, (ii) informed written consent to participate in the study, (iii) age above 21 years, (iv) euthyroidism, (v) Hertel exophthalmometry ≥20 mm, (vi) absence of eye abnormalities such as degenerative myopia, microphthalmos, and anophthalmia, (vii) absence of orbital abnormalities such as previous fractures, surgery, or congenital defects, (viii) absence of eye motility diseases such as myasthenia gravis, and (ix) sufficient cooperation during the evaluation.

### 2.1. Clinical Measurements

The patients were submitted to a complete ophthalmological examination and orthoptic assessment, including a qualitative version evaluation of the nine positions of gaze. A single experienced strabismus specialist made all clinical version assessments.

Versions were graded taking into account basic anatomical landmarks such as the position of the limbus in relation to the medial and lateral canthus (horizontal versions) and the excursion beyond the primary gaze position (vertical versions). We used a scale from −1 to −4 to qualify hypofunction and a scale from +1 to +4 to qualify hyperfunction for each muscle in its field of action. Normal versions were noted as 0 [3, 13–15].

To evaluate the ability of the photographic method to detect different patterns of version impairments and assess the correlation between the two methods (qualitative vs. quantitative), we first classified each version of individual patients with the clinical evaluation and divided the results into three categories:

1. Normal (no hypofunction)
2. Moderate alteration (hypofunction of the evaluated muscle from −1 to −2)
3. Severe alteration (hypofunction of the evaluated muscle from −3 to −4)

### 2.2. Photography

**Figure 1:**
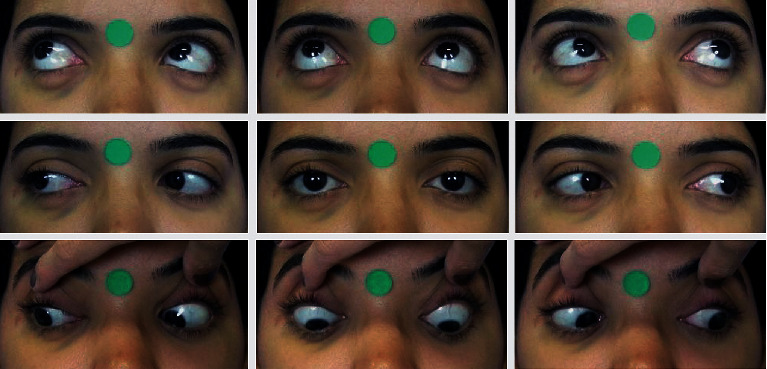
Photographs of the 9 cardinal positions of gaze.

A single trained ophthalmologist took standardized frontal photographs (Canon Power-Shot SX530 HS) of each subject. The patient was positioned in a chair with a clean background at a distance of 50 cm from the camera lens. With the head adequately aligned horizontally and vertically, photographs were taken in the nine cardinal positions of gaze (primary gaze, supradextroversion, supraversion, supralevoversion, dextroversion, levoversion, infradextroversion, infraversion, and infralevoversion). Verbal encouragement was given to ensure head stability and maximum effort toward the extremes of gaze. In case of inappropriate movement, the photographs were repeated. In the infraversions, the eyelids were pulled for better observation. The photograph also included a 12-mm circular sticker for digital calibration (Figure 1).

### 2.3. Digital Photographic Measurements

**Figure 2:**
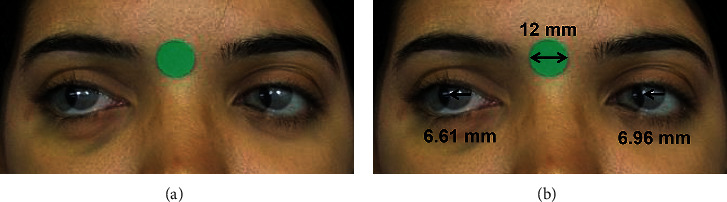
(a) Juxtaposition of semitransparent images of primary gaze and dextroversion (Photoshop) for quantitative version evaluation. (b) Evaluation of dextroversion. Right eye: in abduction, the distance between the medial limbi of the juxtaposed photos is measured. Left eye: in adduction, the distance between the lateral limbi is measured.

A single researcher processed and analyzed the digital images using the method proposed by Lim et al. [11, 16]. Using the software Photoshop (Adobe, San Jose, CA, USA, version 19.1.9), semitransparent photographs of the patient's versions were successively juxtaposed on a photograph in the primary gaze position (Figure 2(a)) [11].

We then measured the distance (mm) between the limbi of the overlapping photographs with the assistance of the software ImageJ (the National Institutes of Health, Bethesda, MD, USA, version 1.52a) [11]. Pixels and mm were calibrated using the 12-mm circular sticker as reference (Figure 2(b)).

As per Lim's method, the limbus-to-limbus distance (mm) was converted into degrees of eyeball rotation with the formula *α* = arcsin (*D/r*), where ɑ is the angle of ocular movement, *D* is the interlimbus distance, and *r* is the external radius of the eyeball, based on axial length measured with the IOLMaster biometer (Zeiss Humphrey System, Dublin, CA, USA) [11].

### 2.4. Statistical Analysis

The statistical analysis was performed using the software Stata v. 15 (StataCorp, College Station, TX, USA) and Statistica v. 13 (TIBCO Software Inc., Palo Alto, CA, USA).

The descriptive statistics included arithmetic means and standard deviations. We used ANOVA or Student's t-test for independent samples to assess the agreement between qualitative and quantitative variables.

We calculated the mean of the maximum angle of the eight secondary and tertiary gaze positions for each clinical category. Using ANOVA and the Tukey-HSD test, we compared the three qualitative categories with regard to the mean angle of version. Statistically significant differences between the means of each category were considered an indication of agreement between the methods.

We also used the Spearman correlation coefficient to assess the correlation between clinical qualitative categories and photographic quantitative measurements.

All statistical tests used an alpha error of 5%. Thus, results were considered statistically significant when *p* < 0.05.

## 3. Results

All 42 patients met the inclusion criteria, with a predominance of the female sex (*n* = 31; 73.8%). The mean age was 48.7 ± 11.9 years.

**Figure 3:**
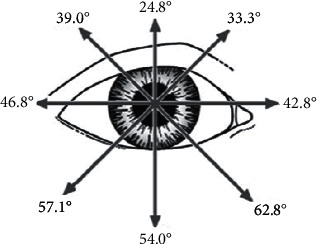
Mean degrees of versions in the cardinal positions of gaze in Graves' orbitopathy patients with clinically normal versions (based on Lim and colleagues) [11].

Figure 3 shows the mean angles of the 8 secondary and tertiary positions of gaze in patients with normal clinical versions.

Tables 1 and 2 show the mean (±standard deviation) measurements (in degrees) of the quantitative measurements for each clinical category. The mean quantitative measures corresponding to the 3 qualitative categories (normal, moderate alteration, and severe alteration) differed significantly in 5 positions of gaze: abduction, adduction, elevation in abduction, elevation, and elevation in adduction (the only two exceptions among the 15 correlations being “normal vs. moderate in abduction” and “moderate vs. severe in elevation in adduction”), indicating a good level of agreement between the two methods (Table 1). On the other hand, in the 3 remaining positions of gaze (depression in abduction, depression, and depression in adduction), which are barely affected in GO, the mean quantitative measures did not vary significantly between the two possible categories (normal vs. moderate alteration) (Table 2).

**Figure 4:**
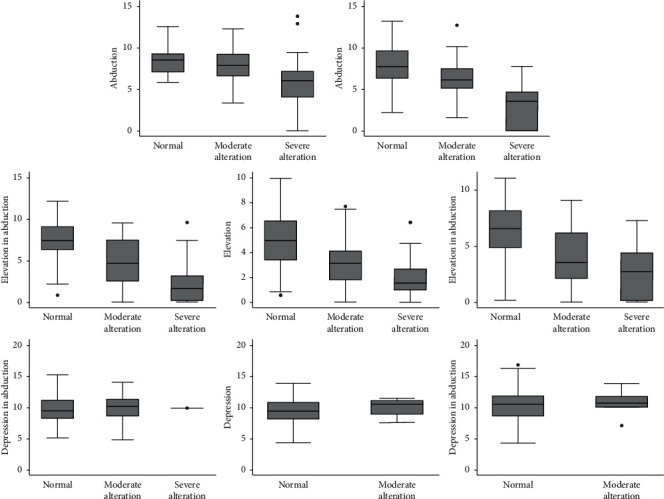
Graphic representation of the agreement between qualitative clinical categories and quantitative photographic measurements of version in the 8 secondary and tertiary positions of gaze in 42 patients with GO.

Figure 4 is a graphic representation of the agreement between the two methods concerning each of the 8 secondary and tertiary positions of gaze.

We also assessed the correlation between the two methods using Spearman correlation coefficients. Statistically significant negative correlations were observed for the following variables: abduction (rho = −0.321, *p* < 0.001), adduction (rho = −0.405, *p* < 0.001), elevation in abduction (rho = −0.627, *p* < 0.001), elevation (rho = −0.527, *p* < 0.001), and elevation in adduction (rho = −0.554, *p* < 0.001). No statistically significant correlations were observed between the methods for depression in abduction (rho = 0.055, *p*=0.477), depression (rho = 0.069, *p*=0.376), and depression in adduction (rho = 0.062, *p*=0.430) (Table 3).

## 4. Discussion

Ductions are termed as uniocular rotations while versions are synchronous simultaneous rotations of the two eyes in the same direction. Version evaluation can identify subtle imbalances in eye movements that may be missed in duction evaluation [2]. Several methods of assessing ocular rotations during the extrinsic eye motility examination have been proposed, but few studies have compared these methods [4, 6, 7, 17]. In 1899, in one of the first studies on eye movement, Asher evaluated his versions [5]. Later, in 1916, Hess recorded the static position of the eyes on a two-dimensional chart (the Hess screen test). The test has since been automated and is currently used to evaluate diplopia and changes in extraocular movements. The Lancaster screen test and the Harms wall test use screens to record eye positions and vertical, horizontal, and torsional deviations [5].

The limbus test was developed by Kestenbaum. He measured ocular versions in millimeters with a transparent ruler positioned in front of the cornea, making it possible to compare the position of the limbus from the primary to the secondary and tertiary positions of gaze [8]. Urist, in turn, developed the lateral version light-reflex test in which the examiner places a luminous focus in front of the patient's eye and observes the position of the light reflex in the sclera while the patient performs extreme lateroversion. The difference is measured in millimeters and converted into degrees using the Hirschberg scale (1 mm = 7°) [9].

Other authors have used ophthalmic devices to measure ductions and versions with greater accuracy. Thus, in 1950, Yamishoro used a keratometer to determine the position of the limbus in adduction, abduction, and supra- and infraduction in a sample of 100 healthy patients [5]. More recently, in 1994, Mourits measured the ductions of 40 healthy patients using a modified Schweiger perimeter [10]. The synotophore may be used to evaluate binocular rotations, despite the 30° limitation in the evaluation of vertical rotations, but the most commonly used ophthalmic device for measuring binocular rotations is Goldmann's manual perimeter [18]. Using a manual perimeter, Haggerty and colleagues concluded that measurements with less than 5° variation might be considered accurate and reliable [18]. Finally, Kushner developed the so-called cervical-range-of-motion device (CROM) to record binocular rotations, anomalous head positions, and binocular field of view [19].

Holmes proposed a photographic method for assessing abduction restrictions in patients with sixth cranial nerve palsy. The method is based on photographs of the patient fixating in dextro- and levoversion. With a ruler, the examiner measures the abduction deficit in millimeters. At the time, the method was considered simple, effective, and reproducible, with good interobserver agreement [20].

More recently, eye-tracking methods or search coils have been used to measure eye movement automatically and quantitatively. However, these methods are too laborious and costly for everyday clinical practice [21].

The techniques discussed above yield highly variable results. Moreover, their usefulness is, in many cases, limited by the need for ophthalmological devices, such as manual perimeters, which are becoming obsolete and can only evaluate ductions.

In the present study, we evaluated the method most commonly employed in clinical practice (qualitative assessment) and a simple and affordable quantitative method of measuring versions based on digital photographs [11].

The qualitative clinical method of version assessment is highly dependent on examiner skill and therefore associated with considerable interobserver variability. This is particularly relevant for patients with GO whose therapeutic follow-up requires quantifying running changes in version amplitude [5].

The digital photographic method of Lim and colleagues is a modification of a method originally proposed by Kestenbaum (the limbus test). Patients are photographed with a digital camera while fixing in the nine positions of gaze. The obtained images are then analyzed with the software Photoshop and ImageJ, and the interlimbus distance (mm) is converted into degrees to determine the maximum angle of movement in each position. Inexpensive and easy to perform, the method is associated with very low interobserver variability (i.e., good reproducibility and accuracy) [11].

The two methods were in agreement concerning five positions of gaze (abduction, adduction, elevation in abduction, elevation, and elevation in adduction). That is, a hypofunction clinically diagnosed as moderate (−1 or −2) correlated well with an angle measured by digitalized photographs that were statistically different from the findings for normal version or severe hypofunction (−3 or −4). Therefore, if performed by an experienced examiner, qualitative and quantitative assessments are likely to yield similar results. However, quantitative photographic assessments are easier to perform, making it possible for different and even less experienced physicians to obtain consistent results at different times, or to remotely diagnose patients, discuss therapies, and monitor response (telemedicine).

The statistical difference observed for the five positions of gaze above was not replicated in the assessment of the infraversions (depression in abduction, depression, and depression in adduction), most likely because in general GO patients are known to display only mild changes in infraversion. Accordingly, in our sample, no cases of severe infraversion alterations were observed and few patients displayed even moderate abnormalities (depression in abduction *n* = 9, depression *n* = 4, and depression in adduction *n* = 6, all of whom with a clinically diagnosed hypofunction of −1). This would explain why the mean quantitative measurements corresponding to the two possible clinical categories (normal and moderate alterations) were not significantly different.

Whether GO is treated surgically or clinically, changes in versions should be measured with the most objective method possible. The quantitative method evaluated in this study yields relatively consistent measurements between examiners and thus is a more useful tool in the evaluation of changes in ocular movement following treatment. However, regardless of the method, the quality of the measurements depends on a wide range of factors: patient comfort, control of head movement, simplicity and accuracy of the procedure, reproducibility, and inter- and intraobserver variability.

The literature provides no gold standard for assessing eye movements. At this point, traditional methods requiring devices that are no longer manufactured (such as manual perimeters) should be replaced. Digital photography appears to be an affordable, reproducible, and accurate alternative.

In conclusion, we found strong correlations between the qualitative clinical method and the quantitative photographic method of assessing ocular versions, especially with regard to lateral and supraversions, which are most typically affected in GO. Ophthalmologists are advised to adopt digital photography for the assessment of ocular versions due to its practicality, suitability for telemedicine applications, and ease of monitoring during follow-up.

## Figures and Tables

**Table 1:** Agreement between qualitative clinical categories and quantitative photographic measurements of version in 5 positions of gaze (abduction, adduction, elevation in abduction, elevation, and elevation in adduction) in a sample of 42 patients with GO.

Version	Qualitative	Quantitative (°) mean (SD)	*p* value^*∗*^	Normal vs. moderate	Normal vs. severe	Moderate vs. severe
				*p* value^Ŧ^	*p* value^Ŧ^	*p* value^Ŧ^
Abduction	Normal (*n* = 18)	46.88 (8.04)	<0.001	0.161	<0.001	0.001
	Moderate alteration (*n* = 52)	42.06 (9.20)				
	Severe alteration (*n* = 14)	31.33 (15.07)				

Adduction	Normal (*n* = 47)	42.84 (10.95)	<0.001	<0.001	<0.001	0.007
	Moderate alteration (*n* = 34)	32.68 (9.20)				
	Severe alteration (*n* = 3)	15.66 (16.26)				

Elevation in abduction	Normal |(*n* = 47)	39.05 (10.95)	<0.001	<0.001	<0.001	<0.001
	Moderate alteration (*n* = 19)	24.20 (15.07)				
	Severe alteration (*n* = 18)	10.36 (10.30)				

Elevation	Normal (*n* = 51)	24.83 (10.36)	<0.001	<0.001	<0.001	0.025
	Moderate alteration (*n* = 18)	15.66 (9.20)				
	Severe alteration (*n* = 15)	8.62 (6.89)				

Elevation in adduction	Normal |(*n* = 52)	33.36 (10.95)	<0.001	<0.001	<0.001	0.242
	Moderate alteration (*n* = 18)	18.05 (12.12)				
	Severe alteration (*n* = 14)	13.29 (10.95)				

^*∗*^ = ANOVA; Ŧ = *post hoc* test (Tukey-HSD). Normal = no hypofunction; moderate alteration = hypofunction from −1 to −2; severe alteration = hypofunction from −3 to −4.

**Table 2:** Agreement between qualitative clinical categories and quantitative photographic measurements of version in 3 positions of gaze (depression in abduction, depression, and depression in adduction) in a sample of 42 patients with GO.

Version	Qualitative	Quantitative (°) mean (SD)	*p* value^*∗*^
Depression in abduction	Normal (*n* = 75)	57.14 (9.78)	0.573
	Moderate alteration |(*n* = 9)	59.31 (12.12)	

Depression	Normal (*n* = 80)	54.09 (9.78)	0.468
	Moderate alteration (*n* = 4)	59.31 (6.89)	

Depression in adduction	Normal (*n* = 78)	62.87 (10.95)	0.268
	Moderate alteration (*n* = 6)	68.43 (8.04)	

^*∗*^ = Student's *t*-test for independent samples. Normal = no hypofunction; moderate alteration = hypofunction from −1 to −2.

**Table 3:** Correlation between qualitative clinical categories and quantitative photographic assessments of version in a sample of 42 patients with GO.

Variable	Rho^*∗*^	*p* value^*∗*^
Abduction	−0.321	<0.001
Adduction	−0.405	<0.001
Elevation in abduction	−0.627	<0.001
Elevation	−0.527	<0.001
Elevation in adduction	−0.554	<0.001
Depression in abduction	0.055	0.477
Depression	0.069	0.376
Depression in adduction	0.062	0.430

^*∗*^ = Spearman correlation coefficient.

## Data Availability

The datasets used and analyzed during the current study are available upon reasonable request to the co-author Allan C. Pieroni Gonçalves (allanpieroni75@gmail.com).
